# Investigating the genetic effects of metformin-related targets on IgA nephropathy using Mendelian randomization

**DOI:** 10.1080/0886022X.2025.2553843

**Published:** 2025-09-03

**Authors:** Yan Zhang, Jinyan Lin, Peizhuang Zheng, Xingtao Lin, Daowen Zheng

**Affiliations:** a Department of Ophthalmology, Zhujiang Hospital, Southern Medical University, Guangzhou, China; b Department of Health Management, Nanfang Hospital, Southern Medical University, Guangzhou, China; c Department of Medical Imaging, Nanfang Hospital, Southern Medical University, Guangzhou, China; d Department of Pathology, Guangdong Provincial People’s Hospital, Guangzhou, China; e Department of Gerontology, Zhujiang Hospital, Southern Medical University, Guangzhou, China

**Keywords:** Immunoglobulin a nephropathy, metformin, Mendelian randomization, ETFDH

## Abstract

The role of metformin and its downstream targets in IgA nephropathy (IgAN) remains unclear. In this study, we used Mendelian randomization to explore potential causal links between genetic proxies of metformin targets, plasma proteins, and the risk of IgAN. Data on plasma protein, metformin target genes, and IgAN data were obtained from the IEU OpenGWAS Project. Single-nucleotide polymorphisms (SNPs) associated with metformin target genes and plasma proteins were selected as instrumental variables. We applied a two-sample and two-step Mendelian randomization approach to examine the causal relationships among these variables. Inverse variance weighted analysis revealed that electron transport flavoprotein dehydrogenase (ETFDH) inhibitors were associated with a reduced risk of IgAN (odds ratio [OR] = 0.948, 95% confidence interval [CI]: 0.923–0.973, *p* < 0.001). A significant causal relationship was identified between ETFDH inhibitors and 592 plasma proteins. Among these, 420 were identified as protective factors. Additionally, 133 plasma proteins were significantly associated with IgAN, of which 78 were identified as risk factors for IgAN. Synaptosome-associated protein 29 was identified as a plasma risk factor for IgAN (OR = 1.118, 95% CI: 1.025–1.218, *p* = 0.012) and negatively correlated with ETFDH inhibitor use (OR = 0.775; 95% CI, 0.709–0.848; *p* < 0.001). This protein appeared to mediate the effects of ETFDH inhibitors on the risk of IgAN (total effect: 0.054; mediation ratio: 52.524%). ETFDH may serve as a potential novel target downstream of metformin-associated metabolic pathways.

## 1. Introduction

Immunoglobulin A nephropathy (IgAN) is the most prevalent form of primary glomerulonephritis worldwide, affecting approximately 2.5 individuals per 100,000 adults annually [1,2]. Despite its high prevalence, the pathogenesis of IgAN remains poorly understood, forcing clinicians to rely exclusively on invasive renal biopsies for definitive diagnosis. The disease carries a substantial burden of morbidity and mortality, with 30–50% of treated patients progressing to renal failure within 10–20 years of disease onset. The median survival time is only 11.4 years, highlighting the critical need for specific biomarkers that enable both early diagnosis and reliable monitoring of disease progression [3,4].

These diagnostic and therapeutic challenges highlight the urgent need for deeper insight into IgAN pathophysiology and the identification of new therapeutic agents and targets. Current treatment options remain limited and often fail to adequately address underlying disease mechanisms, resulting in suboptimal outcomes in many patients.

Metformin, a biguanide widely prescribed for type 2 diabetes, has demonstrated promising renoprotective effects in various nephropathies, particularly diabetic nephropathy [5–8]. Its beneficial effects are mediated through multiple signaling pathways, notably by attenuating oxidative stress, reducing inflammation, and inhibiting apoptotic processes in renal tissues. These mechanistic insights suggest that metformin offers therapeutic benefits beyond its established antidiabetic effects in patients with IgAN. Emerging evidence indicates that the dysregulation of plasma protein profiles may play an important role in IgAN pathogenesis [9–11]. Preclinical studies using murine models of kidney disease have demonstrated that alterations in circulating plasma proteins significantly influence disease trajectory and outcomes [9]. In the clinical setting, proteomic analysis of plasma samples from patients with IgAN has revealed that specific protein modifications are strongly correlated with adverse clinical outcomes [11], highlighting their key role in the pathophysiology of IgAN. Certain plasma proteins are potential diagnostic markers of nephropathy and IgAN [10,11]. Recent studies have shown that metformin alters the levels of various plasma proteins, potentially influencing metabolic pathways associated with diabetes. For instance, metformin therapy reduces the concentration of proinflammatory markers and elevates proteins associated with enhanced insulin sensitivity, thereby improving glycemic control and lowering the risk of diabetes-related complications [12]. Therefore, we proposed that plasma proteins mediate the effects of metformin on IgAN. However, the causal relationship among metformin, plasma proteins, and IgAN pathogenesis remains unclear. Research in this field has predominantly consisted of observational and basic experimental studies that, despite their value, have inherent limitations in establishing causality [13]. This gap in our understanding necessitates more robust methodological approaches to establish the causal links among metformin, plasma proteins, and IgAN pathogenesis.

Mendelian randomization (MR) has emerged as a powerful methodological framework for inferring causality in complex biological systems. This approach leverages genetic variations linked to specific exposures as instrumental variables (IVs) to examine the association between the corresponding phenotype and disease [14,15]. Recent advancements in genome-wide association studies (GWAS) and the identification of molecular mechanisms have strengthened the foundations of MR research.

In this study, we employed a sophisticated two-sample, two-step MR approach to systematically explore the causal correlations among metformin, plasma proteins, and IgAN. This methodology enabled us to investigate the potential therapeutic mechanism of metformin in IgAN and to elucidate the intermediary role of plasma proteins in mediating these effects, offering new insights for targeted therapeutic development.

## 2. Materials and methods

### 2.1. Research design and data sources

**Figure 1.**
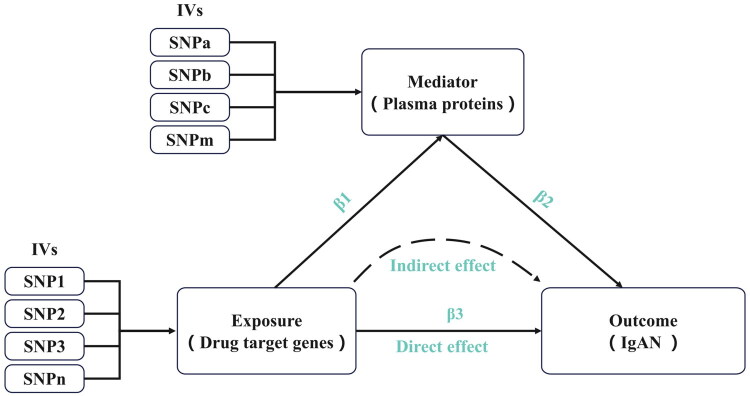
Overview of the study design. β1: MR effect of metformin on plasma proteins; β2: MR effect of plasma proteins on IgAN; β3: MR effect of metformin on IgAN. MR, Mendelian randomization; IgAN, immunoglobulin A nephropathy.

A two-sample MR design was used in this study (Figure 1). To ensure the credibility of potential causal effects, MR analysis must satisfy three fundamental assumptions: (1) genetic variation is strongly correlated with exposure; (2) genetic variation cannot be influenced by confounding factors; and (3) genetic variation influences the outcome only through exposure. Additionally, genetic variations must be located within approximately 500 kb of the cis-acting region of the target gene.

The expression quantitative trait loci (eQTLs) for the metformin target genes *PRKAB1*, *GPD1*, and *ETFDH*, as well as GWAS data for 2992 plasma proteins and IgAN, were obtained from the IEU OpenGWAS project website (https://gwas. mrcieu. ac. uk//). All data were derived from European populations (Table 1). As informed consent was obtained from participants in the original studies, additional ethics committee approval was not required for our analysis. This study followed epidemiological observational studies using the STROBE-MR guidelines [16].

**Table 1.** Summary information on expression quantitative trait loci (eQTLs) and genome-wide association studies (GWAS) databases in Mendelian randomization (MR) studies.

Data source	Phenotype	Sample size	Cases	Population
eqtl-a-ENSG00000111725	PRKAB1	31684	–	European
eqtl-a-ENSG00000167588	GPD1	30836	–	European
eqtl-a-ENSG00000171503	ETFDH	26395	–	European
IEU Open GWAS project	Plasma proteins	3301	–	European
ebi-a-GCST90018866	IgAN	477784	15587	European

### 2.2. Setting the conditions of IV

To establish appropriate instrumental variables (IVs), we filtered single nucleotide polymorphisms (SNPs) using a significance threshold of *p* < 1 × 10^−5^ and required an F-statistic greater than 10 to identify and eliminate weak IV.

### 2.3. Screening of IVs for metformin target gene eQTLs

A linkage disequilibrium (LD) coefficient of r^2^ = 0.3, linkage imbalance region width of 100 kb, and minimum secondary allele frequency (MAF > 0.01) were set to ensure the independence of the SNPs and eliminate the impact of linkage imbalance on the results. Single SNPs linked to confounding factors and outcomes were eliminated using PhenoScanner. We extracted IVs within 500 kb of the cis-regulatory region of the drug target gene from the eQTLs data of the drug target gene. The relevant SNPs were extracted from the GWAS summary data for the outcome variables (IgAN and plasma proteins). SNPs with a palindromic structure and MAF > 0.42 were excluded, as SNPs were directly associated with the outcome variables (IgAN and plasma protein) at (*p* < 5 × 10^−8^).

### 2.4. Screening of plasma protein IV

For plasma protein IVs, we set the LD coefficient r^2^ to <0.001, and the width of the linkage imbalance region to 10,000 kb. This ensured that each SNP was independent, thereby eliminating the influence of a linkage imbalance. The PhenoScanner was used to exclude single SNPs associated with confounders and outcomes. Relevant SNPs were extracted from the pooled GWAS data for the outcome variable (IgAN). SNPs with a palindromic structure, MAF > 0.42, and SNPs directly related to the outcome variable (IgAN) were excluded (*p* < 5 × 10^−8^).

### 2.5. Statistical and analysis methods

#### 2.5.1. Causal association analysis

Five regression models were used: Mendelian randomization-Egger (MR-Egger) regression, random-effects inverse variance weighted (IVW), weighted median, weighted mode, and simple mode, with IVW as the primary analysis method. When the number of SNPs was ≤ 3, the effect of a single SNP on the outcome was measured using the Wald ratio; otherwise, the fixed-effect IVW method was applied. When > three SNPs were included, the random-effects IVW method was used. In the causal effect estimation based on multiple SNPs as IV, the reciprocal of the variance (R^2^) of each locus was used as the weight. The causal effect estimates for each locus were weighted and summed using the IVW method. For the horizontal pleiotropy assessment, we referred to the results of the MR-Egger study.

#### 2.5.2. Heterogeneity test method

The heterogeneity of SNPs was assessed using Cochran’s Q test. Statistical significance was set at *p* < 0.05, indicating heterogeneity. I^2^, another measure of heterogeneity, represents the proportion of total variation attributed to it. The I^2^ values range from 0% to 100%. An I^2^ value of >50% indicated the presence of heterogeneity in the IVW results.

#### 2.5.3. Pleiotropy analysis method

The intercept terms of the MR-Egger and MR-PRESSO methods were used for pleiotropy analysis. A nonzero intercept in the MR-Egger regression model (*p* > 0.05) indicated that the IVs were not pleiotropic. Similarly, a *P*-value > 0.05 in MR-PRESSO indicates that the IVs are not pleiotropic.

#### 2.5.4. Sensitivity analysis method

The leave-one-out method was employed for sensitivity analysis, in which each SNP was removed individually and the remaining SNPs were reanalyzed to assess the effect of removing each SNP on the overall analysis results.

All analyses were performed using the two-sample MR package in R 4.1.0, with a significance level α of 0.05. The RMediation package was used to compute the 95% CI for the mediation effect.

#### 2.5.5. Mediating effects analysis

The total effect of exposure on the outcomes can be divided into direct and indirect (mediated) effects. This study used two-step MR analysis to explore the mediating effects of metformin-induced plasma proteins on IgAN. The mediated effect was calculated as β1 × β2, where β1 is the MR effect of metformin on plasma proteins and β2 is the MR effect of plasma proteins on IgAN. The proportion (E%) of the mediating effect was calculated using the formula (β1 × β2)/β3, where β3 represents the total effect of metformin on IgAN.

## 3. Results

From the eQTLs data of the metformin drug target genes (*PRKAB1*, *GPD1*, and *ETFDH*), 45, 0, and 33 SNPs associated with IgAN were identified, respectively (Additional File 1). In total, 59,607 SNPs were identified in the eQTLs data for *ETFDH*, a metformin target gene (Additional File 2). Additionally, 32,697 SNPs associated with IgAN were obtained from plasma protein analysis (Additional File 3).

### 3.1. Causal association analysis of metformin drug target genes with IgAN

The IVW results showed that the PRKAB1 activator was not significantly associated with the risk of IgAN (odds ratio [OR] = 0.975, 95% confidence interval [CI]: 0.948–1.002, *p* = 0.074). The IVW results demonstrated that ETFDH inhibitors were associated with a reduced risk of IgAN (OR = 0.948, 95% CI: 0.923–0.973, *p* < 0.001). The IVW results revealed no heterogeneity between ETFDH inhibitors and eQTLs associated with IgAN (I^2^ = 0%; Cochran’s *Q* = 28.753; *p* = 0.632; Table 2). The MR-Egger results indicated no significant disparity between the intercept term and 0 (*p* = 0.137), and the *P*-value of MR-PRESSO was 0.679, suggesting the absence of horizontal pleiotropy in the SNPs and confirming the robustness of the MR findings in this study.

**Table 2.** Causal association results of mendelian randomization (MR) regression between metformin and immunoglobulin a nephropathy (IgAN).

Exposure	Method	OR (95% CI)	*p*	I ^(Nihei^ et al.^2023)^ (%)	Cochran’s Q	*p*	Egger intercept	SE	*p*	MR-PRESSO
PRKAB1 Activator	IVW	0.975 (0.948–1.002)	0.074	37	69.611	0.008	–	–	–	0.008
	MR-Egger	0.988 (0.946–1.033)	0.602	37	68.603	0.008	−0.004	0.005	0.431	0.008
	Simple mode	0.927 (0.859–1.000)	0.058	–	–	–	–	–	–	0.008
	Weighted median	0.956 (0.923–0.990)	0.011	–	–	–	–	–	–	0.008
	Weighted mode	0.966 (0.933–0.999)	0.052	–	–	–	–	–	–	0.008
ETFDH Inhibitor	IVW	0.948 (0.923–0.973)	7.5 *E* − 05	0	28.753	0.632	–	–	–	0.679
	MR-Egger	0.982 (0.931–1.036)	0.514	0	26.423	0.701	0.009	0.006	0.137	0.679
	Simple mode	0.976 (0.922–1.034)	0.420	–	–	–	–	–	–	0.679
	Weighted median	0.959 (0.924–0.996)	0.029	–	–	–	–	–	–	0.679
	Weighted mode	0.963 (0.926–1.001)	0.065	–	–	–	–	–	–	0.679

Note: The exposure variables in the table are metformin genes for different drug targets and the outcome variable is IgAN; SE: standard error.

### 3.2. Causal association of plasma proteins with IgAN and ETFDH inhibitors

The eQTLs of ETFDH were used to estimate the causal effect of ETFDH inhibitors on plasma proteins. The IVW method demonstrated that the causal association between ETFDH inhibitors and 592 plasma proteins was significant (Additional File 4). Among these, 420 were protective factors, such as synaptosomal-associated protein 29 (SNAP29) (OR = 0.775, 95% CI: 0.709–0.848, *p* < 0.001), and 172 were risk factors, such as melanoma B antigen 3 (OR = 1.289, 95% CI: 1.152–1.441, *p* < 0.001). The ETFDH inhibitor acted as a protective factor against the plasma protein SNAP29 (OR = 0.775, 95% CI, 0.709–0.848; *p* < 0.001). IVW results indicated no heterogeneity between the ETFDH inhibitor and plasma SNAP29-related SNPs (I^2^ = 34%, Cochran’s *Q* = 18.193, *p* = 0.110). The MR-Egger results revealed no significant differences in the intercept term and 0 (*p* = 0.929), and the *P*-value of MR-PRESSO was 0.101, indicating no horizontal pleiotropy in the SNPs and confirming the robustness of the results.

Genetic variations associated with plasma proteins were used to assess the causal impact on IgAN susceptibility. The IVW method revealed a statistically significant causal relationship between 133 plasma proteins and IgAN (Additional File 5). The plasma protein SNAP29 emerged as a risk factor for IgAN development (OR = 1.118, 95% CI: 1.025–1.218, *p* = 0.012). No heterogeneity was observed between the plasma protein SNAP29 and the SNPs associated with IgAN (I^2^ = 23%, Cochran’s *Q* = 7.816, *p* = 0.252). The MR-Egger results indicated no significant deviation from zero for the intercept term (*p* = 0.483), and the *P*-value of MR-PRESSO was 0.318, suggesting the absence of horizontal pleiotropy in the SNPs and confirming the robustness of the MR findings.

### 3.3. Evaluation of the mediating effect of plasma proteins in ETFDH inhibitors and IgAN

**Figure 2.**
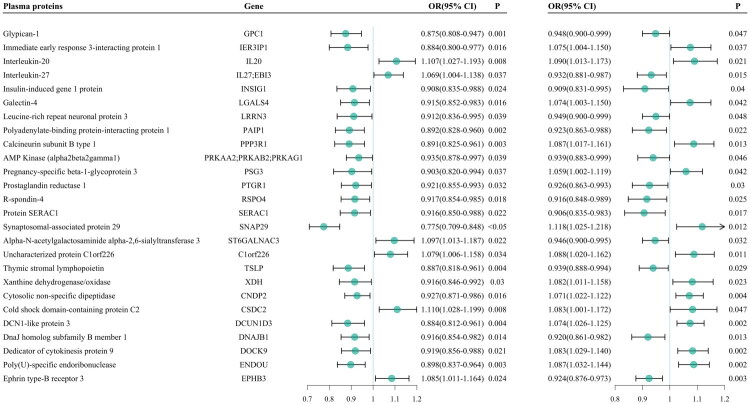
Forest plot of the effect of ETFDH inhibitors on plasma proteins and the effect of plasma proteins on IgAN. ETFDH, electron transport flavoprotein dehydrogenase; IgAN, immunoglobulin A nephropathy.

Twenty-six plasma proteins showed significant causal associations with the ETFDH inhibitors and IgAN (Figure 2). ETFDH inhibitors act as protective factors for 20 plasma proteins, including SNAP29, and as risk factors for six plasma proteins, including cold shock domain-containing C2. Additionally, 13 plasma proteins, including serine active site-containing protein 1, were identified as protective factors against IgAN pathogenesis, while 13 plasma proteins, including SNAP29, were identified as risk factors for IgAN pathogenesis.

Thirteen plasma proteins exhibited mediating effects (Table 3). Among these proteins, SNAP29 mediated the effect of an ETFDH inhibitor on IgAN, with an overall effect of β = −0.054, a mediating effect of β = −0.028, and a mediating ratio of 52.524% (95% CI: 50.08%–54.968%). The indirect effect of the ETFDH inhibitor on IgAN was estimated based on the overall and mediating effects (β = −0.026).

**Table 3.** The intermediary effect of ETFDH inhibitors on immunoglobulin a nephropathy (IgAN) *via* plasma proteins.

Mediation	Mediation.Gene	Mediating effect	Direct effect	Total effect	Proportion mediated (%)
Synaptosomal-associated protein 29	SNAP29	−0.028	−0.026	−0.054	52.524(50.080–54.968)
Calcineurin subunit B type 1	PPP3R1	−0.010	−0.044	−0.054	17.960(16.930–18.989)
Poly (U)-specific endoribonuclease	ENDOU	−0.009	−0.045	−0.054	16.548(15.718–17.377)
Immediate early response 3-interacting protein 1	IER3IP1	−0.009	−0.045	−0.054	16.507(15.347–17.667)
DCN1-like protein 3	DCUN1D3	−0.009	−0.045	−0.054	16.470(15.613–17.326)
Xanthine dehydrogenase/oxidase	XDH	−0.007	−0.047	−0.054	12.835(11.930–13.740)
Dedicator of cytokinesis protein 9	DOCK9	−0.007	−0.047	−0.054	12.416(11.678–13.154)
Ephrin type-B receptor 3	EPHB3	−0.006	−0.047	−0.054	12.006(11.275–12.737)
Galectin-4	LGALS4	−0.006	−0.048	−0.054	11.738(10.904–12.573)
Pregnancy-specific beta-1-glycoprotein 3	PSG3	−0.006	−0.048	−0.054	10.837(10.006–11.667)
Cytosolic nonspecific dipeptidase	CNDP2	−0.005	−0.049	−0.054	9.704(9.131–10.276)
Alpha-N-acetylgalactosaminide alpha-2,6-sialyltransferase 3	ST6GALNAC3	−0.005	−0.049	−0.054	9.457(8.789–10.126)
Interleukin-27	IL27; EBI3	0.005	−0.049	−0.054	8.739(8.129–9.350)

## 4. Discussion

In this study, we examined the causal relationship between metformin target genes and IgAN from a genetic perspective and investigated the role of plasma proteins in mediating this association. Our findings indicated that an ETFDH inhibitor, a target gene of metformin, is associated with a lower risk of IgAN. Our analysis revealed a significant association between ETFDH inhibitors and 592 plasma proteins, of which 420 were protective and 172 were risk factors. We established significant causal links between 133 plasma proteins and IgAN, with SNAP-29 identified as a risk factor for IgAN. Furthermore, 26 plasma proteins showed significant causal associations with ETFDH inhibitors and IgAN. Thirteen plasma proteins exhibited mediating effects, with SNAP-29 being the most significant mediator of the effects of ETFDH inhibitors on IgAN.

Our findings suggest that the inhibition of ETFDH, a key enzyme in mitochondrial fatty acid oxidation, is associated with a reduced risk of IgAN, which may be mediated by the modulation of immune cell function and inflammatory pathways. Studies have indicated that ETFDH can influence cellular energy levels by regulating mitochondrial function and oxidative phosphorylation [17], which may be closely associated with chronic inflammatory responses and immune system functions, which are particularly in patients with IgAN. Additionally, animal models of IgAN treated with an ETFDH inhibitor exhibited improved renal histology and function compared with the control group, indicating its beneficial effects on kidney health. These findings emphasize ETFDH as a potential new target downstream of the Metformin-related pathway. Recent studies suggest that Metformin may indirectly affect the ETFDH-related pathway through metabolic reprogramming[18,19], but it does not directly pharmacologically inhibit ETFDH itself. It also highlights the necessity to further explore its mechanisms in human studies to validate preclinical results and assess its translational potential[20,21].

Our research indicates that ETFDH inhibition protects SNAP29, a protein crucial for vesicle transport and autophagy, which are vital for homeostasis and immune cell function [22]. SNAP29 has been identified as a risk factor for IgAN, suggesting a complex interplay among ETFDH inhibition, SNAP29 levels, and immune cell activity. Two-step MR analysis revealed that ETFDH inhibition exerts a protective effect on IgAN through SNAP29, with a substantial mediating effect. This indicated that SNAP29 may serve as a critical mediator in the pathway linking ETFDH inhibition to reduced IgAN risk, potentially through its effects on immune cell function and inflammatory responses.

SNAP29 is a soluble NSF attachment protein receptor (SNARE) that plays a crucial role in cell membrane fusion and autophagy, particularly in the degradation of intracellular substances by facilitating autophagosome-lysosome fusion. For instance, RUN domain-containing protein 1, which interacts with autophagy-related protein 14, negatively regulates autophagy by blocking syntaxin 17-SNAP29-vesicle associated membrane protein 8 complex formation, thereby preventing autophagosome-lysosome fusion [23]. Microtubule-associated protein 1 A/1B-light chain 3, lysosomal-associated membrane protein 2, and immunity-related GTPase family M proteins assist syntaxin 17 in autophagosomes [24]. In patients with IgAN, the expression levels of autophagy-related genes, including microtubule-associated protein 1 A/1B-light chain 3, and p62, are significantly elevated and positively correlated with disease severity. These findings suggest that autophagy contributes to the pathology of IgAN and affects disease progression by modulating inflammation and fibrosis [25,26].

However, the specific roles of SNAP29 and ETFDH in IgAN require further investigation. Their importance in endomembrane fusion, autophagy, and energy metabolism offers new perspectives for understanding this disease. The dysfunction of these molecules may contribute to immunoglobulin A (IgA) deposition and inflammation through various mechanisms that exacerbate disease progression. Therefore, targeting the ETFDH-SNAP29 axis may offer a novel mechanistic target for modulating immune cell activity and reducing inflammation in patients with IgAN.

It should be particularly noted that the causal inference derived from MR reflects the lifelong and systematic effects produced by genetic variation, which is fundamentally different from the dose-dependent, reversible, and tissue-specific effects of drug interventions such as Metformin. Therefore, the downregulation effect of ETFDH obtained through genetic proxy variables in this study should not be directly interpreted as a pharmacological effect, but rather regarded as a potential biological mechanism worthy of further exploration. This limitation is an inherent characteristic of the methodology of MR, which has been discussed in detail in previous literature [27].

This study presents genetic evidence for the positive effects of ETFDH on IgAN through the regulation of plasma proteins. Despite its robustness, this study had several limitations. First, this study relied only on bioinformatics and statistical analyses, and there is no direct evidence that wet laboratory experiments can elucidate the biological mechanisms. Secondly, although the sample size was obtained from a large GWAS dataset, it may have been inadequate to detect subtle genetic effects or interactions. In subsequent studies, we will directly explore the specific biological mechanism of ETFDH inhibition of plasma proteins and IgAN using cellular and animal models. By expanding the sample size, more samples from different demographics and ethnicities could be collected to improve the ability to detect subtle genetic effects or interactions. Another limitation of this study is that we were unable to evaluate the recognized target GPD1 of metformin due to the lack of valid instrumental variables. No GPD1 expression-related SNPs were found to meet our screening threshold, making MR analysis of this gene impossible. This limitation limits our ability to fully capture the lineage of metformin molecules. Given the important role of GPD1 in the regulation of gluconeogenesis, future studies using more comprehensive transcriptomics or tissue-specific eQTL datasets may lead to the discovery of new genetic tools to explore the role of GPD1-related pathways in IgAN and other diseases.

## 5. Conclusions

This study reveals potential metabolic pathways associated with the risk of IgAN, indicating that ETFDH may serve as a potential novel target downstream of metformin-associated metabolic pathways. This mechanism may be partially mediated by the regulation of SNAP29 and its effects on immune cell function. Further research is needed to elucidate the precise mechanisms by which SNAP29 influences the immune response in IgAN and to explore the therapeutic potential of targeting this pathway in a clinical setting.

## Supplementary Material

supplementary table X.xlsx

Supplementary files.xlsx
